# Effects of PTFE Micro-Particles on the Fiber-Matrix Interface of Polyoxymethylene/Glass Fiber/Polytetrafluoroethylene Composites

**DOI:** 10.3390/ma11112164

**Published:** 2018-11-02

**Authors:** Jasbir Singh Kunnan Singh, Yern Chee Ching, De Shin Liu, Kuan Yong Ching, Shaifulazuar Razali, Seng Neon Gan

**Affiliations:** 1 Department of Chemical Engineering, Faculty of Engineering, University of Malaya, 50603 Kuala Lumpur, Malaysia; jasbi@siswa.um.edu.my; 2 Department of Mechanical Engineering, Faculty of Engineering, University of Malaya, 50603 Kuala Lumpur, Malaysia; shaiful@um.edu.my; 3 Department of Mechanical Engineering, Faculty of Engineering, National Chung Cheng University, Chiayi County 62102, Taiwan; imedsl@ccu.edu.tw; 4 School of Foundation, University of Reading Malaysia, Persiaran Graduan, Kota Ilmu, Educity, 79200 Iskandar Puteri Johor, Malaysia; kuanyong84@hotmail.com; 5 Department of Chemistry, Faculty of Science, University of Malaya, 50603 Kuala Lumpur, Malaysia; gansn@yahoo.com

**Keywords:** coefficient of friction, wear, surface etch, PTFE, POM, interface

## Abstract

Reinforcing polyoxymethylene (POM) with glass fibers (GF) enhances its mechanical properties, but at the expense of tribological performance. Formation of a transfer film to facilitate tribo-contact is compromised due to the abrasiveness of GF. As a solid lubricant, for example, polytetrafluoroethylene (PTFE) significantly improves friction and wear resistance. The effects of chemically etched PTFE micro-particles on the fiber-matrix interface of POM/GF/PTFE composites have not been systematically characterized. The aim of this study is to investigate their tribological performance as a function of micro-PTFE blended by weight percentage. Samples were prepared by different compositions of PTFE (0, 1.7, 4.0, 9.5, 15.0 and 17.3 wt.%). The surface energy of PTFE micro-particles was increased by etching for 10 min using sodium naphthalene salt in tetrahydrofuran. Tribological performance was characterized through simultaneous acquisition of the coefficient of friction and wear loss on a reciprocating test rig in accordance to Procedure A of ASTM G133-95. Friction and wear resistance improved as the micro-PTFE weight ratio was increased. Morphology analysis of worn surfaces showed transfer film formation, encapsulating the abrasive GF. Energy dispersive X-ray spectroscopy (EDS) revealed increasing PTFE concentration from the GF surface interface region (0.5, 1.0, 1.5, 2.0, 2.5 µm).

## 1. Introduction

Polymer composites are physical mixtures of a polymer, known as the matrix, and a reinforcing filler, called the dispersed phase. The latter is added to enhance mechanical, tribological or other properties [1]. Thermoplastics and thermosets are the two types of matrix used in this class of composites. The reinforcing fillers can be organic or inorganic, in the form of fibers or particles. The important types of polymer-based composites (PBCs) contain fibers, particles, or a combination of both [2]. The matrix and reinforcement are separated by an interphase, a term introduced in the 1970s [3]. Karger-Kocsis et al. [4] reviewed the recent advancements in fiber/matrix interphase tailoring of fiber-reinforced polymer composites. This study involves composites of polymer matrices blended with fibers and particles as reinforcing fillers. 

The use of thermoplastics as the matrix for PBCs has an advantage because formation can be carried out by injection molding, compression molding, or extrusion techniques. These processes are very economical for manufacturing components that require good precision, low cost, and high volume. As such, PBCs are widely used in automotive, aviation, marine, and construction industries. 

Fibers are used as a reinforcement to improve mechanical properties such as tensile strength and elasticity modulus. Decreased fiber dimension lowers the probability of flaws or imperfections, thus making them significantly stronger and stiffener than a matrix. The matrix transfers the load to these fibers and stresses are distributed among them. Stress transferred from matrix to fiber and fiber to fiber is dependent on the interphase. The matrix also allows positioning of fibers and protects the fibrous reinforcement from the environment in which these composites are used. 

Fiber-reinforced PBCs are widely used in tribological designs owing to their light weight, and excellent mechanical, self-lubricating, and wear resistance properties. These polymer composites are usually grouped based on the type of reinforcing fibers and matrices. The appearance of these reinforcing fibers can be classified as discontinuous, continuous, or aligned. They are further categorized into organic and inorganic. High strength inorganic fibers such as glass (GF), carbon (CF), and ceramic fibers are used to improve mechanical properties. In this paper, we studied the tribological properties of polyoxymethylene/glass fiber/polytetrafluoroethylene (POM/GF/PTFE) composites on a reciprocating test rig. This test method utilized a flat POM composite specimen and a stainless-steel ball as the upper specimen that slides against the stationary composite.

Polymer tribology is vastly different from traditional tribology, which was originally developed for metals. The main differences between polymers are the viscoelasticity, time dependent properties and absorption of liquid lubricants [5,6]. It is important to understand polymer tribology and select an appropriate test methodology to simulate the service life of components produced using PBCs [7]. Polymeric materials manufacturers usually maintain confidentiality on the actual composition of resins. Therefore, designers depend on catalogue information and academic literature when selecting the appropriate material to be used in applications. The vast amount of dispersed and scattered data force designers to conduct their own lab-scale experiments to gain confidence in their design solution [8,9]. 

POM is an engineering plastic with outstanding tribological properties and a good balance of mechanical and thermal properties. It has good self-lubricating characteristics with a low coefficient of friction and high wear resistance [10,11]. However, use of pure POM is limited to conditions of low sliding speed and low load. By depending on its own inherent properties, pure POM may not be appropriate in applications requiring superior mechanical and tribological properties [12,13,14]. Therefore, these properties must be improved to extend its range of applications. Numerous studies have reported the development of POM composites as self-lubricating materials in applications related to engineering, automotive, bearings, electronic appliances, and building materials [15,16]. This has been achieved by blending with other polymers, fibers, and micro- or nano-sized particles. These modifiers were organic and non-organic [17,18].

The blending of GF as reinforcement has been one of the approaches to improve tensile strength and elasticity modulus. Higher strength and stiffness are achieved as a result of the strength of GF holding the POM matrix together and the bond of GF to the POM matrix. The stability of the fiber-matrix interphase determines the change of these properties [19,20]. The GF acts as an effective reinforcement, on the condition that its adhesion to the matrix is good. When impacted or loaded, the energy absorbed by the GF makes the polymer tougher and stronger. This is noticeable when the morphology of the fractured surfaces of filled versus reinforced polymers is compared after impact testing [21,22]. However, wear resistance and friction coefficient are negatively affected by the addition of GF to POM [23].

PTFE is often added as a filler in polymers to reduce friction and wear. Other advantages include its resistance to organic and inorganic solvents, its hydrophobic properties, and its electrical and thermal insulating capabilities. In PBCs, the disadvantage of using PTFE as a reinforcing phase is its low free surface energy. This causes weak molecular interactions between the composite components. The free energy of PTFE can be increased by either coarsening its surface or creating new functional chemical groups on the surface layer. This task has been fulfilled through surface modification to enhance its compatibility to the matrix. Chemical etching, electron beam irradiation, or plasma treatment [24,25] are among the methods commonly employed to alter its mechanical and/or chemical structure.

According to Drzal’s concept [26], the interphase is a three-dimensional region between the bulk fiber and bulk matrix. It is the two-dimensional area of contact between the fiber and the matrix, known as the interface, and a region of finite thickness extending in both sides of the fiber and matrix interface [27]. Interactions between filler and matrix are dependent on the interphase, which determines the mechanical properties. During the preparation of polymer composites, the wetting by liquid polymers on solid surfaces and the adhesion forces must be considered [28]. For PBCs, different approaches have been adopted to modify the interphase to enhance its properties [29,30].

The load transfer capability of the interphase depends on fiber and matrix adhesion. It can be physico-chemical, frictional, or both [31,32,33,34]. Physico-chemical adhesion, comprising chemical reactions [35,36] and intermolecular interactions [37,38], is more important than frictional adhesion obtained, by example, by surface roughening [39]. Practically, these components may be at work simultaneously. Effective load transfer capability along with abrasion resistance is required in applications where mechanical and tribological properties are of equal importance.

In recent years, modification of fiber-matrix interphase via incorporation of micro- or nano-sized particles has been investigated widely. The approach has an advantage, as the properties are altered without any change of processing conditions. Well-dispersed particulate fillers fit between fibers, improving the interfacial shear strength and, thereby, the mechanical properties of fiber-reinforced composites. However, the influence of these particles on the fiber-matrix adhesion has shown inconsistent results. Arao et al. [40] reported improved mechanical properties by incorporating nanofillers into carbon fiber/polypropylene (PP) matrix. The interfacial shear strength remarkably improved between GF and PP by dispersing expanded graphite nanoplatelets (xGnP) based on the investigation by Pegoretti et al. [41]. On the contrary, Zhang et al. [42] found that inclusion of nano-silica particles in carbon fiber/epoxy did not affect the interfacial bonding behavior between fibers and the matrix. In these types of three-phase composites, only the matrix-dominated properties were improved. 

Kumar et al. [43] developed a micromechanical model to predict the stress transfer through the interphase of fiber-reinforced composites. Shear and radial stresses at the interphase provide insight for the design of engineered interfaces/interphases. To characterize the interphase of fiber-reinforced composites, different approaches have been reported. Cech et al. [27] successfully determined a region of 0.5 µm thickness in the periphery of glass fiber using atomic force microscopy (AFM) and dynamic mechanical analysis (DMA). A nano-scratch method reported by Schoneich et al. [44] distinguished the fiber, matrix, and the interphase layer. Olmos et al. [45] revealed gradual phase separation at the interphase depending on the distance of glass fiber surface and epoxy modified with polymethylmethacrylate (PMMA) matrix. The PMMA domains were removed by immersing in dichloromethane (CH_2_Cl_2_) for one day before scanning electron microscope (SEM) morphology and calculation of PMMA particle density analysis from the fiber surface. Other indirect methods, such as the pull-out test, the microbond test, and the single fiber fragmentation test were reviewed by Graupner et al. [46].

Significant research has been dedicated to develop POM composites to achieve a low frictional coefficient and better wear resistance, as well as excellent tensile strength and elasticity modulus. Li et al. [47] studied the effects of nano- and micro-PTFE particles on the tribological properties of POM. Transfer films played an important role in stabilizing the coefficient of friction, but at the expense of mechanical properties. Benabdallah [48] evaluated the friction coefficient and wear on POM composites using a reciprocating sliding motion against steel surfaces with two different coatings. POM/GF was more abrasive whereas POM/PTFE demonstrated better tribological properties through transfer film formation. Zhang et al. [49] systematically experimented with properties of reinforcing fillers on the transfer film structure of POM-fiber composites. Addition of silica nanoparticles (SiO_2_) to POM/GF did not ameliorate the tribological properties but blending PTFE micro-particles into POM/GF significantly enhanced the coefficient of friction and wear. Evidence of a uniform PTFE-based transfer film formation on the counter surface was reported.

In this work, POM/GF was used as the matrix where GF acts as the reinforcement phase. The composition of GF was unchanged at 25% by weight ratio. The surface of micro-PTFE was etched for 10 min using sodium naphthalene salt in tetrahydrofuran. The PTFE micro-particles were then melt-blended into the matrix. The effects of these surface etched micro-PTFE on the fiber-matrix interface were studied by characterizing the coefficient of friction, wear loss, morphology of worn surfaces, and the chemistry of the interface layer. The aim of this work was to characterize the tribological properties of POM/GF/PTFE composites as a function of micro-PTFE content. There have been studies on tribological performance of POM composites blended with various reinforcing fillers. Based on a literature search, the effects of surface etched micro-PTFE on the fiber-matrix interface of POM/GF/PTFE composites have not been systematically studied. In our previous work [50], optimal tensile strength, elasticity modulus, toughness, and hardness for POM/GF/PTFE composites was achieved when the content of micro-PTFE was 6.5% and etch time was 10 min.

## 2. Materials and Methods

In this study, POM with 25% GF reinforcement used as matrix material was purchased from Du Pont. POM is a homopolymer commercially known as POM525GR with density of 1.6 g/cm^3^ and melting temperature of 178 °C. PTFE micro-particles used as solid lubricant with an average diameter, density, and specific area of 12 µm, 0.425 g/cm^3^,and 1.5–3.0 m^2^/g, respectively, was also procured from Du Pont. The etch solution was prepared in the lab by dissolving sodium naphthalene salt in tetrahydrofuran obtained from J.T. Bakker. The sodium salt’s density was 0.45 g/cm^3^.

Preparation of the etch solution was carried out using a magnetic stirrer. Sodium naphthalene salt was added to tetrahydrofuran at 25 °C with 350 rpm stirring speed. The mixture, comprising 5% sodium naphthalene to 95% tetrahydrofuran, was stirred for 5 min. A dark brown saturated solution was formed. Next, 30 g of PTFE micro-particles was added to the etch solution and stirred at 25 °C for 10 min with stirring speed of 525 rpm. Upon completion of the stirring cycle, the sediments were left to settle for about 1 min. The solid sediment, comprising sodium salt and micro-PTFE, settled at the bottom. The upper liquid was then poured away carefully. 

The sediment was subjected to a wash cycle using 200 cm^3^ of acetone. The mixture was stirred for 5 min at 525 rpm. Upon completion, the sediment was left to settle for 3 min. The upper liquid was discharged before subjecting the residue to two further wash cycles. All wash cycles utilized the same volume of acetone and stirring conditions.

The solid was then rinsed in 200 cm^3^ of distilled water. A total of 5 rinse cycles were conducted by stirring for 2 min with stirring speed of 525 rpm. After each rinse cycle, the solid was left to settle for 3 min. The upper portion, consisting of dissolved sodium salt in distilled water, was poured away before repeating the next rinse cycle. These rinse cycles effectively separated the PTFE micro-particles from the sodium salt. The slurry PTFE micro-particles were then poured into a 150 mm diameter petri dish that formed a layer approximately 1 mm thick. The petri dish was placed in an incubator maintained at 40 °C for 48 h to remove the water. Lastly, the dry micro-PTFE was removed from the petri dish, transferred into a lab container and stored in a dark environment to prevent exposure to light. SEM and Fourier Transform Infrared Spectroscopy (FTIR) techniques were employed to characterize the micro-PTFE etched for 0 min, 10 min and 17.1 min. 

Melt blending was carried out using a Brabender Mixer 50EHT 3Z (Brabender GmBH & Co KG, Kulturstraße, Duisburg, Germany) to blend POM525GR and surface etched micro-PTFE. The mixer’s processing parameters were temperature, blade rotational speed, and mix time. These parameters were maintained at 180 °C, 60 rpm and 10 min, respectively. The blend was then crushed to approximately 1–3 mm in length. POM/GF/PTFE composite samples were prepared by injection molding process using a BOY XS machine (BOY Machines, Inc., Exton, PA, USA). The molding comprised three main processes, i.e., filling, plasticizing, and holding. The filling process required injection pressure of 14 MPa with injection speed of 100 mm/s. For plasticizing process, pressure, screw rotational speed, and barrel temperature were controlled to 1 MPa, 170 rpm and 180 °C, respectively. During injection of the melt into the mold, holding pressure of 12 MPa was maintained.

Tribology tests were performed using a Ducom Reciprocatory Friction and Wear Monitor (Ducom Instruments, TR-281-M8, Bangalore, India) according to Procedure A of ASTM G133-95, unlubricated wear testing. POM composite samples of 12.5 × 12.5 × 4.0 mm^3^ were placed on the stationary stage. The counterpart was an stainless steel sheet, SST 440C (Grade 24) stainless steel ball of 4.7625 mm radius mounted to the reciprocating arm. Normal load of 25 N was applied using dead weights. The stroke length and oscillating frequency was controlled to 10 mm and 5 Hz. Each sample was tested for 1000 s resulting in sliding distance of 100 m. Table 1 shows the POM composites with varying PTFE content to determine coefficient of friction and wear loss measured directly on the Ducom tester. The reported data are mean values of three replicated tests.

All contacting surfaces were cleaned using a cloth dampened with isopropyl alcohol (IPA) to remove presence of any contamination. Tests were performed under ambient laboratory conditions with relative humidity (RH) of 50 ± 5% and temperature maintained at 22 ± 2 °C. During reciprocating sliding motion, variations in the normal load occurred because of vibrations and inertia effects. The data acquisition system provided simultaneous and real time measurement of normal and friction forces at 5 Hz frequency. A new SST 440C ball was used for every test. Upon test completion, the POM composite samples were carefully removed from the test rig. Each sample was individually placed in a 70-mL capped polypropylene lab container to prevent any contact to the tested surface.

Surface morphology of worn surfaces for POM composites was inspected using an electronic microscope (Keyence Corporation, VHX-500, Osaka, Japan). Further examination of these surfaces was performed under a Phenom ProX (Phenom-World B.V., Eindhoven, The Netherlands) desktop Field Emission Scanning Electron Microscope (FE-SEM). SEM microscopy was also employed to determine effects of chemical etching on the micro-PTFE.

Fiber-matrix mapping for presence of fluorine atoms at the interface region was carried out using Quanta FEG-450 (Thermo Fisher Scientific, Hillsboro, OR, USA) SEM. The geometry of the sample was a type IV dumbbell shape prepared using injection molding. Gauge-length section of the dumbbell was milled out to a specimen of 3 mm (L) × 3 mm (W) × 10 mm (H). The 9 mm^2^ surface area was ground using silicon carbide (Si-C) paper with a grain size from 600 to 1200 before applying gold sputtering. Samples were placed on a motorized stage located inside a vacuum chamber. The roomy chamber enabled navigation of samples in three axes for optimal view and analysis. The Quanta SEM system, equipped with electron backscatter diffraction (BSD) and energy dispersive X-ray spectroscopy (EDS), was operated at 15 kV. The AZtec analysis software package (Oxford Instruments Nano-Analysis, High Wycombe, UK) allowed elemental identification at specific points, at 0.5 µm intervals from the GF surface.

Fourier transform infra-red (FTIR) spectroscopy was performed utilizing the PerkinElmer Spectrum 400 FTIR spectrometer unit (PerkinElmer, Waltham, MA, USA) using the KBr pellet technique with the resolution of 4 cm^−1^ and 32 scans per recording. The functional groups of chemically etched PTFE micro-particles were compared against non-etched sample. POM/GF/PTFE composites were further characterized for presence of any new functional groups as a result of nucleophilic reaction.

## 3. Results

### 3.1. Effects of Chemical Etching on PTFE Micro-Particles

**Figure 1:**
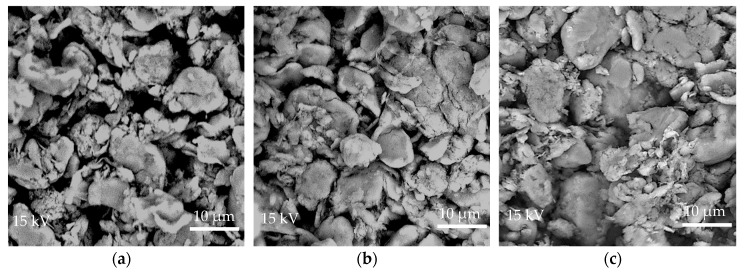
SEM micrographs of micro-PTFE etched for (**a**) 0 min; (**b**) 10 min; and (**c**) 17.1 min.

Figure 1a–c shows comparison of surface morphology between non-etched, 10 min etched, and 17.1 min etched samples of the solid lubricant using SEM under 5200× magnification. The non-etched micro-PTFE displayed a smooth surface and spherical shapes of different sizes. The chemically etched sample exhibited coarser surface morphology. For the 10 min etched sample, a slightly rougher surface was visible on large and mid-sized particles, signifying evidence of surface etching. Clusters of smaller particles were noticeable, likely the remains of disintegrated larger particles that experienced greater effects of chemical etching. As the etch time was increased to 17.1 min, higher etch depth was achieved on the surface and more large particles appeared to be fragmented. In addition, the micro-PTFE appeared to be more densely packed compared to the 10 min and non-etched samples.

### 3.2. Tribological Behavior of POM/GF/PTFE Composites

The Ducom tester allowed instant acceleration and deceleration of the steel ball sliding on stationary POM composites. The dynamic coefficient of friction was computed as the ratio of friction and normal force measured simultaneously. The reciprocating test set up permitted concurrent wear loss measurement.

#### 3.2.1. Friction

**Figure 2:**
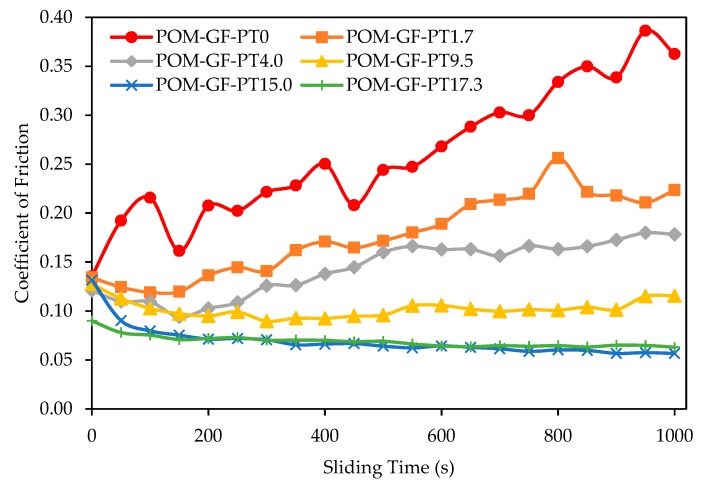
Characteristics of frictional coefficient as a function of sliding time for various POM/GF/PTFE composites.

Tribological properties were remarkably enhanced with the addition of micro-PTFE. The change of frictional coefficient as a function of time for different POM/GF/PTFE composites is shown in Figure 2. At the start of testing, a lower frictional coefficient was registered for composites with higher micro-PTFE content. As the test cycles progressed, the composites displayed either an increasing, stable, or slightly decreasing frictional coefficient depending on the amount of micro-PTFE. Neat POM/GF demonstrated the highest coefficient of friction, that continuously increased throughout the test cycles due to the abrasive GF. A steadily increasing coefficient of friction was also observed for composites with 1.7% and 4.0% PTFE, but at a slower rate compared to neat POM/GF. For composite with 9.5% micro-PTFE, the frictional coefficient stabilized throughout the test. As the micro-PTFE content was further increased to 15.0% and 17.3%, slightly decreasing frictional behavior was observed.

#### 3.2.2. Wear Loss

**Figure 3:**
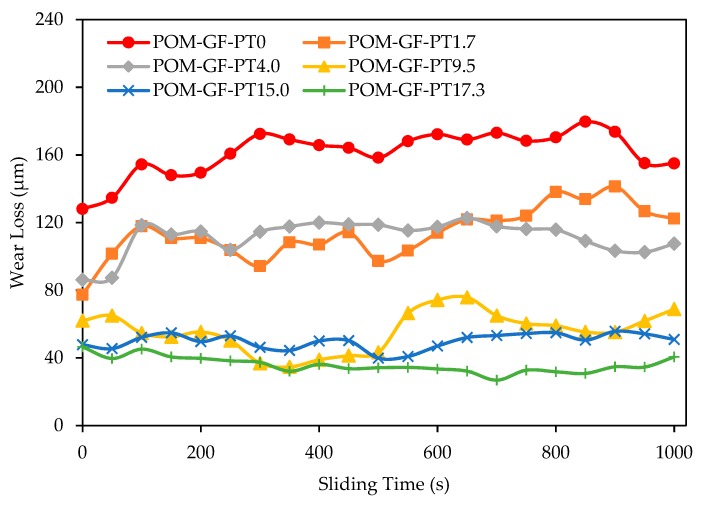
Characteristics of wear loss as a function of sliding time for various POM/GF/PTFE composites.

The test set up allowed simultaneous acquisition of wear loss and coefficient of friction over the test cycles. Considering the curved contacting surface of the steel ball, assumptions were made that the wear scars were flat and the depth of these scars were considered a measurement of wear loss. Figure 3 displays wear loss as a function of sliding time. The reported data were obtained based on three replicated tests. The cyclic behavior of wear loss profiles might be attributed to several factors, some of which include non-homogenous material properties of POM composites, variation of sample roughness during the reciprocating motion of the stainless-steel ball, and inherent system vibration. [12,14]. 

Similar to the effects seen on frictional behavior, the initial wear loss was higher for composites with lower PTFE content. A step increase of wear loss could be observed after 50 s for POM composites with 0%, 1.7%, and 4.0% micro-PTFE. The former two composites also displayed obvious cyclical wear loss patterns. POM composites blended with 9.5%, 15.0%, and 17.3% micro-PTFE registered slightly decreasing wear loss trends. After 500 s, rapid wear loss could be observed for the composite with 9.5% micro-PTFE, whereas a gradual increase was noted for composite with 15.0% micro-PTFE. Further increase in the micro-PTFE content to 17.3% exhibited negligible wear loss throughout the test duration.

### 3.3. Morphology of Worn Surfaces

The worn surfaces were characterized through optical and SEM microscopy under 500× magnification. Optical micrographs offered a wide view of the worn surface morphology whereas SEM microscopy revealed the effects of PTFE micro-particles in detail.

#### 3.3.1. Optical Microscopy of POM/GF/PTFE Worn Surfaces

**Figure 4:**
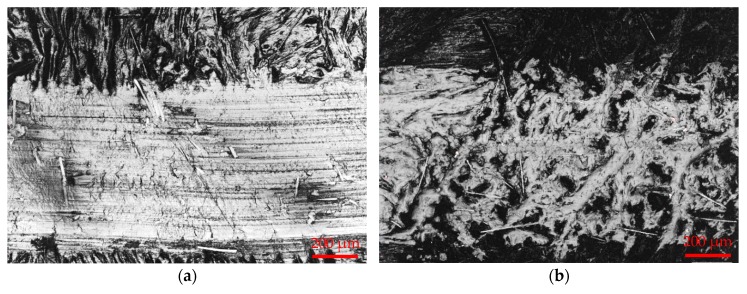

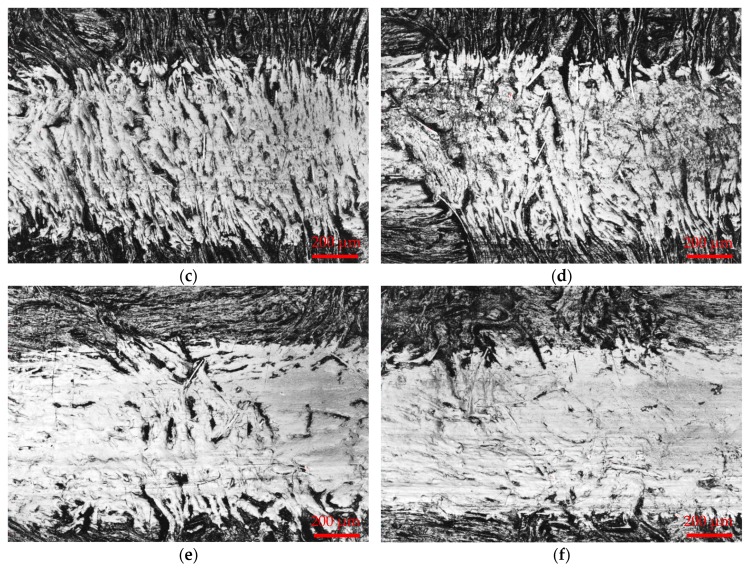
Optical micrographs of POM/GF/PTFE composite worn surfaces after tribology test: (**a**) POM-GF-PT0; (**b**) POM-GF-PT1.7; (**c**) POM-GF-PT4.0; (**d**) POM-GF-PT9.5; (**e**) POM-GF-PT15.0; (**f**) POM-GF-PT17.3.

Figure 4a–f illustrates the wear morphologies of neat POM/GF and POM/GF/PTFE composites with different composition of micro-PTFE. The PTFE micro-particles influenced the morphology of worn surfaces significantly. As depicted in Figure 4a, deep scratch grooves can be observed in the sliding direction due to the abrasiveness of GF. In addition, surface cracks in the normal direction to sliding because of adhesive wear were visible for the neat POM/GF. The composites containing lower micro-PTFE (Figure 4b–d) exhibited obvious wear scars compared to composites with higher PTFE content (Figure 4e,f). Exposed GF on the worn surfaces was obvious for the neat POM/GF and composites with lower PTFE composition. 

The formation of a PTFE-based transfer film is represented by the density of white regions on the worn surfaces. As depicted in Figure 4b–f, the spots of white areas were the flattened peaks, whereas the dark regions are valleys that formed surface asperities. Shear in the contact between the steel ball and composite sample during sliding caused wear debris to fill up the valleys. This led to the formation of a patchy transfer film for POM composites blended with 1.7%, 4.0%, and 9.5% micro-PTFE, and a more homogenous film for composites with higher micro-PTFE composition. 

As the weight percentage of micro-PTFE was increased, the transfer film encapsulated the GF surface along the sliding path. Formation of thicker transfer film resulted in wear loss reduction. The transfer film effectively concealed the valleys and encapsulated the GF, preventing asperities from further damaging the composite material. As noted in Figure 4e,f, continuous and coherent transfer films were formed for composites blended with 15.0% and 17.3% micro-PTFE. These observations explain the lower coefficient of friction and wear loss during tribology testing.

#### 3.3.2. SEM Microscopy of POM/GF/PTFE Worn Surfaces

**Figure 5:**
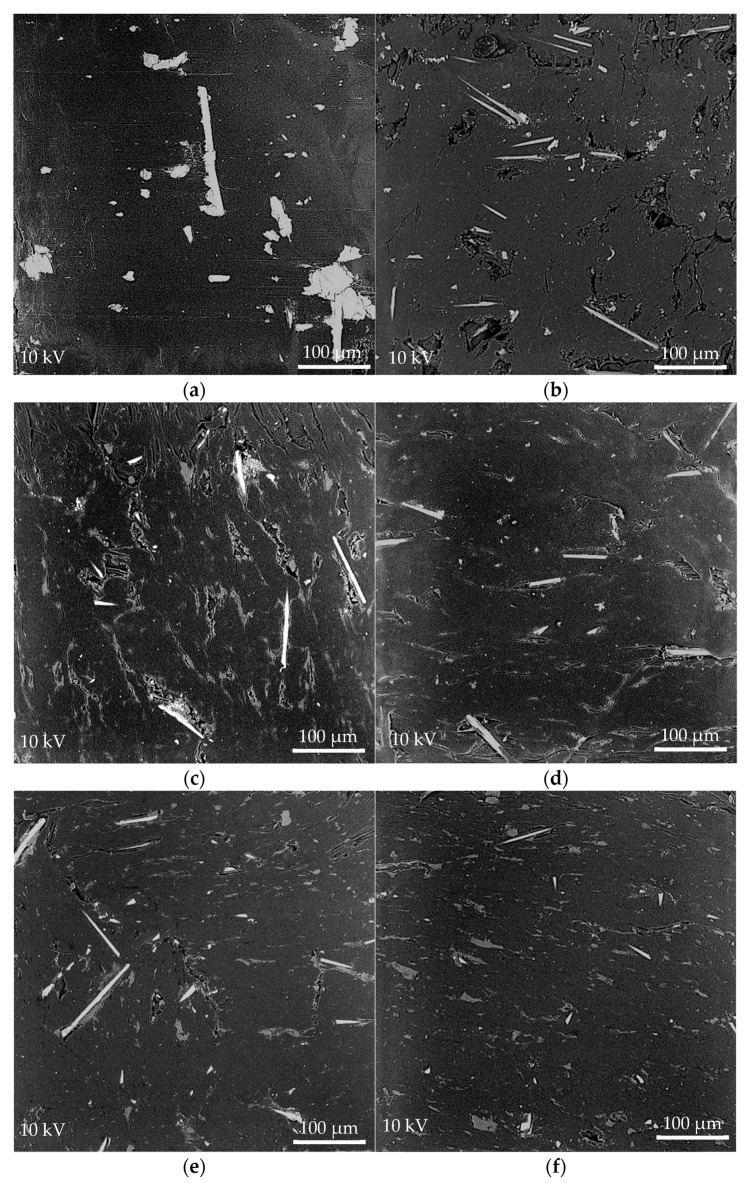
SEM micrographs of POM/GF/PTFE composites worn surfaces after tribology test: (**a**) POM-GF-PT0; (**b**) POM-GF-PT1.7; (**c**) POM-GF-PT4.0; (**d**) POM-GF-PT9.5; (**e**) POM-GF-PT15.0; (**f**) POM-GF-PT17.3.

Figure 5a–f shows the SEM images of worn surfaces for neat POM/GF and its composites. For the neat POM/GF (Figure 5a), continuous reciprocating rubbing of steel ball with high contact pressure caused the GF to be damaged. Fragments of GF, observed as white specks on the SEM micrographs, were generated. These fragments were not only present at the vicinity of GF, but also carried slightly further away from the GF. Similar to optical images, many scratch grooves were observed parallel to the sliding direction. These scuff marks can be seen originating from the damaged GF. The surface temperature increased as a result of friction heat generated during the sliding motion, causing adhesive wear and plastic deformation. High shear stress destroyed the POM, forming surface cracks in the normal direction of sliding. Consequently, both coefficient of friction and wear loss continuously increased.

With the addition of micro-PTFE, stable rubbing conditions were facilitated through formation of a PTFE-based transfer film. As the micro-PTFE content was increased, not only was lesser damage observed on the worn surfaces, but the surfaces also demonstrated the capability to self-repair. The composite blended with 17.3% PTFE (Figure 5f) displayed the smoothest worn surface. The GF and its fragments were fully embedded in the matrix. For composites with lower PTFE contents of 1.7% and 4.0% (Figure 5b,c), damaged regions around the GF remain unrepaired. Formation of an effective transfer film was prevented at these regions due to the abrasiveness of GF and insufficient PTFE. The composites blended with 15.0% and 17.3% micro-PTFE (Figure 5e,f) exhibited a uniform PTFE rich layer, effective to endure the scrapping of hard GF and its fragments. The formation of a smooth surface exhibited a low frictional coefficient and wear loss [51]. As shown in Figure 2 and Figure 3, the composites comprising high PTFE content demonstrated self-repairing capability. 

The composite with 9.5% micro-PTFE formed a relatively smooth surface (Figure 5d). The transfer film effectively coated some, but not all, of the GF. The coefficient of friction and wear loss remained stable up to 500 s into the test before increasing from its steady state condition. It is known that addition of PTFE reduces the strength and stiffness of these composites. Optimal mechanical properties were obtained by blending POM/GF with 6.5% PTFE by weight percentage [50]. In applications where both mechanical and tribological properties are of equal importance, the PTFE amount can be a vital determination factor in order to satisfy the requirement.

### 3.4. FTIR Spectroscopy

Morphology analysis of worn surfaces established that the micro-PTFE amount and its ability to form a uniform transfer film determined the tribological characteristics. The abrasive GF was effectively coated by a PTFE rich layer. In order to clarify the effects of chemically etched micro-PTFE on tribological properties of POM/GF/PTFE composites, a FTIR technique was employed.

#### 3.4.1. Characterization of Surface Etched PTFE

**Figure 6:**
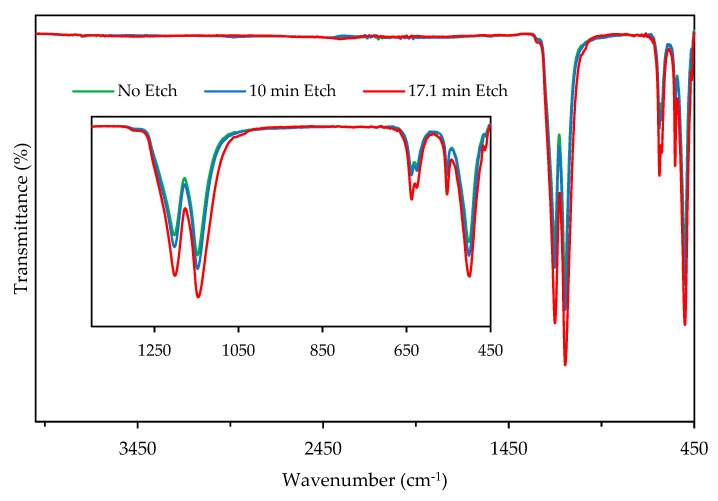
Fourier transform infra-red (FTIR) transmittance of PTFE micro-particles non-etched, 10.0 min etched and 17.1 min etched.

Figure 6 shows the effects of chemical etching by comparing non-etched, 10.0 min etched, and 17.1 min etched PTFE micro-particles studied via FTIR analysis. In the FTIR spectrograms, consistent absorption bands of 501 cm^−1^, 554 cm^−1^, 638 cm^−1^, 1145 cm^−1^, and 1199 cm^−1^ in the C–F region were observed. The first three wave numbers correspond to CF_2_ rocking, CF_2_ bending, and CF_2_ wagging whereas 1145 cm^−1^ and 1199 cm^−1^ can be attributed to the CF_2_ symmetric stretching vibration modes [52,53]. Absence of any new absorption bands within the wave number range indicated nucleophilic substitution of fluorine as a result of chemical etching did not occur.

#### 3.4.2. Characterization of POM/GF/PTFE Composites

**Figure 7:**
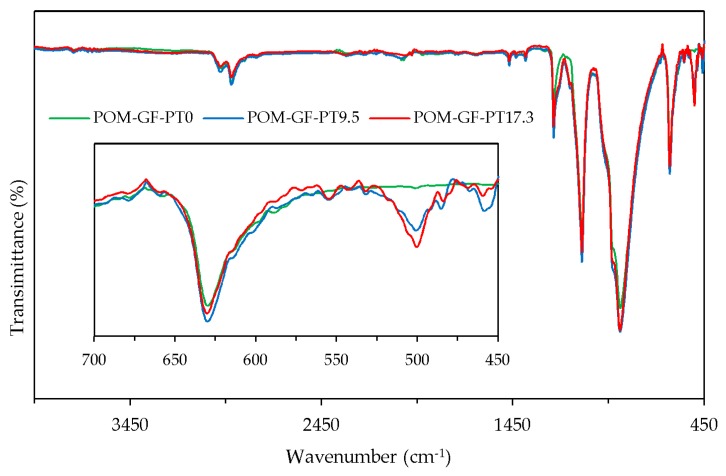
Fourier transform infra-red (FTIR) transmittance of POM/GF/PTFE composites blended with 0%, 9.5% and 17.3% PTFE micro-particles.

In order to study the effects of blending micro-PTFE and POM/GF, FTIR analysis was performed to compare POM/GF/PTFE composites blended with 0%, 9.5%, and 17.3% micro-PTFE (Figure 7). The spectrum exhibited intense integrated bands at 630 cm^–1^ (CH bending), 887 cm^−1^ (COC symmetric stretching), 1089 cm^−1^ (COC symmetric stretching) and 1236 cm^−1^ (CH_2_ rocking). Other peaks at 1470 cm^−1^ (CH_2_/CH_3_ deformation), 2921 cm^−1^ (CH asymmetric stretching), and 2978 cm^−1^ (CH_2_ asymmetric stretching) were attributed to the different vibration modes of groups in the POM chain [54]. The strong absorption band at 501 cm^−1^ (CF_2_ rocking) is assigned to C–F group in PTFE. Absence of new functional groups indicated chemical reaction did not occur. The adhesion mechanism between the polymer melt to chemically etched micro-PTFE was strictly mechanical interlocking, promoted by the frictional component as a result of the roughened surface.

### 3.5. Mapping of Fiber-Matrix Interface Region

**Figure 8:**
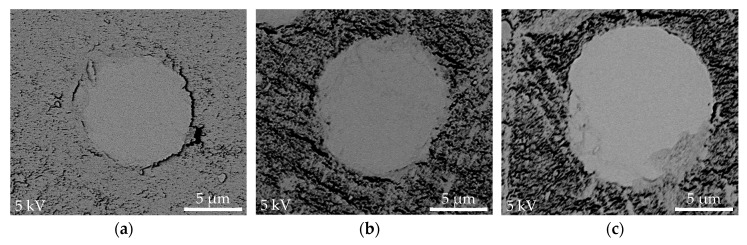
SEM micrographs of the interface and points for elemental analysis using EDS: (**a**) POM-GF-PT1.7; (**b**) POM-GF-PT9.5; (**c**) POM-GF-PT17.3.

**Figure 9:**
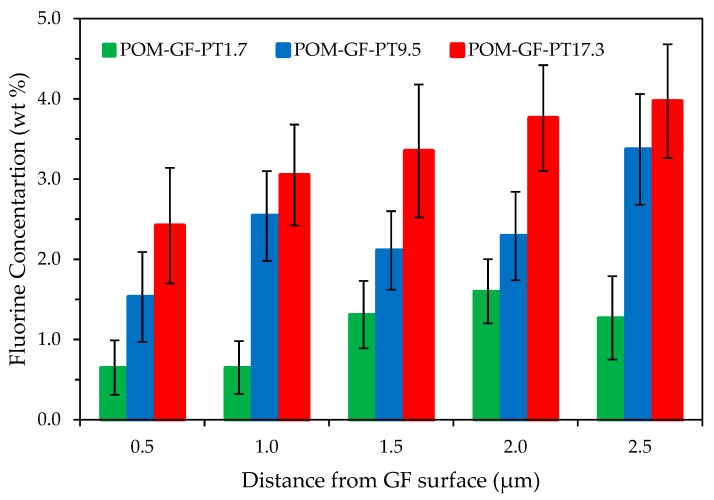
Weight percentage of fluorine atoms as a function of distance from GF surface.

The polished specimens containing 1.7%, 9.5%, and 17.3% micro-PTFE were examined using SEM under 15,000× and elemental analysis using EDS. As shown in Figure 8 and Figure 9, mapping of the composite phases and interface evaluation was carried out on the basis of quantifying weight percentage of fluorine atoms at 0.5 µm intervals away from the GF surface. All composites displayed a gradual increase of fluorine atom fraction further away from GF edge. Composites blended with higher amount of micro-PTFE revealed higher concentration of fluorine atoms. This greatly enhanced the tribological performance by enabling formation of PTFE based transfer film.

## 4. Discussion

This paper investigated the effects of blending PTFE micro-particles in a POM/GF matrix to enhance the tribological properties. As the weight fraction of micro-PTFE was increased, significant reduction of the coefficient of friction and wear loss was observed, supporting the fact that PTFE is an efficient solid lubricant. It was also established that addition of PTFE created low-friction film between the sliding partners, which reduced adhesion [10,49]. For the case of neat POM/GF, POM-based transfer film could not form on the steel ball counter face during the sliding action due to the abrasiveness of GF. 

Similarly, reduction of wear loss was clarified by the improved lubricating properties, attributable to the PTFE micro-particles. Transfer film formed between the POM/GF/PTFE composites and steel ball during the reciprocating motion. As the PTFE content was increased, this transfer film repaired the worn surfaces resulting in either minimal or stable wear loss as witnessed by the SEM micrographs of composites with 9.5%, 15.0%, and 17.3% micro-PTFE. Furthermore, chemical etching of micro-PTFE particles roughened the surface and possibly increased its surface energy. The PTFE rich wear debris effectively filled the scratches, forming an even and dense transfer film. This resulted in better interaction between the transfer film and counter surface, eventually reducing the coefficient of friction and wear loss [49]. The cyclic behavior of wear loss is assumed to be an unstable transfer film thickness dependent on PTFE amount in composites. The non-homogenous material properties of POM composites, variation of sample roughness during the reciprocating motion, inherent system vibration, etc., might be some of the contributing factors [12,14].

Based on the optical micrographs of neat POM/GF, absence of PTFE in the composite and the abrasive GF prevented formation of a homogenous transfer film to facilitate steady lubricating conditions. Black areas surrounding the exposed GF might be caused by damaged polymeric carbon chain due to high pressure and temperature. These conditions caused a sharp increase of the frictional coefficient as the sliding cycles progressed. Addition of micro-PTFE produced more wear debris that filled up surface asperities represented as the dark regions [9]. 

PTFE easily shears to form transfer films due to its molecular structure, resulting in its superior self-lubricating properties. This is caused by the weaker van der Waals force between its molecular chains than the intramolecular bonds. SEM micrographs revealed self-repairing capability of the damaged surfaces because an even and tenacious transfer film was formed. The surface, comprising POM and GF, was coated by this transfer film from direct counterface contact. The wear mechanism was both abrasive and adhesive; that is, mainly abrasive for composites with no or lower PTFE content and primarily adhesive when the composites were blended with a higher PTFE amount. Consequently, the characteristics of the frictional coefficient and wear loss correlated well to the weight percentage of micro-PTFE.

The prolongation of chemical etching led to the increase in the intensity of the absorption bands. The position of these absorption peaks did not shift. These consistent and active centers can be regarded as a non-occurrence of PTFE surface oxidation because of chemical etching [55,56]. Surface morphology analysis using SEM revealed formation of rougher and more porous cavities as the etch time was increased. Disintegration of the micro-particles were also noticeable [50,57]. These physical changes slightly increased PTFE concentration resulting in the higher intensity of FTIR absorption peaks as a function of etch time. The porous defluorination layer promoted mechanical interlocking as the melt blend filled these surface imperfections. Studies performed by Hunke et al. [24,58] showed functional groups in the defluorinated layer were not removed, even at temperatures exceeding 300 °C, enabling surface modified PTFE particles to be used as a tribological property modifier in high-temperature engineering plastics. 

Fiber-matrix interface mapping using the SEM-EDS method was neither able to ascertain a sharp matrix-fiber interface nor determine interphase dimension. However, it adequately established the presence of PTFE at the interface region of the fiber-matrix, altering the tribological properties of POM/GF/PTFE composites. Several researchers [44,45,46] have studied the interphase thickness and identified a value of between 0.03 µm and 3 µm, dependent upon fiber fraction, type of matrix material, and methods used. Experimental techniques of higher precision progressively decreased the interphase thickness.

## 5. Conclusions

In this study, friction and wear behavior of POM composites filled with GF and PTFE micro-particles were comprehensively investigated. In particular, the study examined the mechanism of PTFE to effectively enhance tribological properties through transfer film formation. In the absence of PTFE, stress induced during a reciprocating motion fractured the GF, inducing damage to the POM surface. The abrasive GF prevented formation of a POM-based transfer film. Addition of PTFE greatly enhanced the tribological properties through formation of a PTFE-based transfer film that was capable of enduring the scraping of GF. The worn surfaces, as a result of abrasive and adhesive wear, were self-repaired as the PTFE content was increased. The surface etched PTFE enabled better adhesion to POM through mechanical interlocking. No formation of new function groups was confirmed via FTIR spectroscopy. PTFE, detected as fractions of fluorine atoms at the fiber-matrix interface region, gradually increased in concentration further away from to the GF surface.

## Figures and Tables

**Table 1:** The composition of POM/GF/PTFE composites investigated.

Sample Name	PTFE Etch Time (min)	PTFE Weight (%)
POM-GF-PT0	-	0
POM-GF-PT1.7	10	1.7
POM-GF-PT4.0	10	4.0
POM-GF-PT9.5	10	9.5
POM-GF-PT15.0	10	15.0
POM-GF-PT017.3	10	17.3
